# Developing a Machine Learning Model for Hydrogen Bond Acceptance Based on Natural Bond Orbital Descriptors

**DOI:** 10.1021/acs.joc.5c00724

**Published:** 2025-07-07

**Authors:** Diego Ulysses Melo, Leonardo Martins Carneiro, Mauricio Domingues Coutinho-Neto, Paula Homem-de-Mello, Fernando Heering Bartoloni

**Affiliations:** Centro de Ciências Naturais e Humanas, 74362 Universidade Federal do ABC, Santo André, São Paulo 09210-580, Brazil

## Abstract

This study employs machine learning (ML) to assess the predictive power of electronic descriptors derived from natural bond orbital (NBO) analysis for hydrogen bond acceptance. Using a data set of 979 hydrogen bond complexes, each formed by a hydrogen bond acceptor and 4-fluorophenol as the donor, we optimized geometries via GFN2-xTB, followed by DFT single-point calculations. From these, NBO analysis was used to extract intramolecular donor–acceptor interactions, particularly the orbital stabilization energies (*E* ^(2)^), which reflect electron delocalization and relate to canonical resonance structures. The *E* ^(2)^ values served as features to train seven ML models, based on different techniques: KNN, Decision Tree, SVM, RF, MLP, XGBoost, and CatBoost. To our knowledge, this is the first work that uses *E* ^(2)^ as a standalone ML descriptor for hydrogen bond acceptance. Even with a small set of descriptors, we achieved high predictive performance, with errors below 0.4 kcal mol^–1^, surpassing previous studies that used heterogeneous descriptors, including quantum-chemical data. Our results highlight the utility of NBO-based features in building accurate, physically meaningful, and generalizable ML models for p*K*_BHX_ prediction.

## Graphical abstract

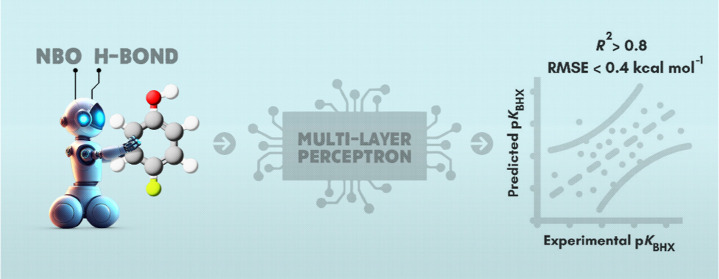

## 1 Introduction

Noncovalent interactions (NCIs) regulate the chemical, physical, and biological properties of a series of compounds.^1^ Among the NCI, which include π···π, lone pair···π, and ion···π interactions, one of the most relevant and widely discussed is the hydrogen bond (H-bond). An H-bond is a short-range interaction that involves dipole–dipole attraction, where a hydrogen bond donor (HBD), comprising a hydrogen atom covalently bonded to a more electronegative atom, interacts and forms an H-bond with a hydrogen bond acceptor (HBA), which is another electronegative atom in the same or at a different molecule.^2^ This interaction plays a particularly crucial role in many biological and molecular systems, such as protein structure,^3^ biological recognition,^4^ and photochemical processes.^5^

Traditionally, it is easy to recognize HBDs when a hydrogen atom bonds to an electronegative nitrogen, oxygen, or fluorine atom. However, molecules with hydrogen bonded to carbon (C–H), phosphorus (P–H), and sulfur (S–H) can also function as H-bond donors.^6–9^ On the other hand, while the number of atoms a hydrogen atom can interact with to form an HBD is limited, there are more flexible conditions to turn a molecule into an HBA. These are related to the presence of lone electron pairs, systems rich in π-electrons, such as those with double and triple bonds and aromatic systems, and hydrogen atoms from metal hydrides, which can also function as HBAs.^10,11^ As such, the list of potential HBAs includes atoms such as carbon, sulfur, fluorine, phosphorus, and hydrides from arsenic, selenium, and transition metal complexes.^12–16^

Although electrostatic interaction is well-recognized as a driving force in H-bond formation, it is necessary to consider other components that can characterize and affect this interaction to explain experimental and theoretical results.^17^ As discussed by van der Lubbe et al.,^18^ these additional components include charge–transfer interactions, π-resonance assistance, steric repulsion, cooperative effects, dispersion interactions, and secondary electrostatic interactions. Additionally, due to the importance of H-bonds in many fields, both experimental and primarily theoretical efforts have been made to quantitatively evaluate and predict the strength of H-bonds, and the complex nature of these interactions has stimulated ongoing research on this topic over the decades.^19–27^

In an intermolecular system, the attractive interaction between an HBA and an HBD molecule, represented by eq 1, leads to the formation of a hydrogen bond complex (HBA···HBD), where···represents the H-bond interaction itself. The experimental determination of the complexation strength involved in forming such a complex is a way of measuring the H-bond acceptance.

1 HBA+HBD⇌HBA···HBD

More recently, machine and deep learning methods have been employed to predict various molecular, materials, and reaction properties.^28–32^ In this work, we hypothesize that the electronic effects arising from the interaction between HBA and HBD can be explored by machine learning (ML) models capable of predicting H-bond acceptance according to the widely used p*K*_BHX_ hydrogen bond basicity scale. While many ML models use a combination of experimental properties and data derived from the theoretical calculation as features for ML, here, we propose using only the values of orbital stabilization energy derived from the natural bond orbital as features for ML regression studies. To the best of our knowledge, the prediction of p*K*_BHX_ by ML strategies, particularly employing natural bond orbital (NBO) based descriptors, has no precedent. In the next sections, we discuss both the p*K*_BHX_ hydrogen bond basicity scale as well as the NBO descriptor.

## 2 Background

### 2.1 p*K*_BHX_ Hydrogen Bond Basicity Scale

The magnitude of the HBA···HBD interaction was initially studied by Gurka et al. through the determination of the formation constant (*K*_f_) using the NMR chemical shift of ^19^F atom from 1:1 mixtures of various HBA molecules with 4-fluorophenol (4-FPh), the latter serving as an HBD.^33,34^ Taft et al. were pioneers in establishing the p*K*_HB_ scale from the logarithm of *K*_f_.^35^ Subsequent research expanded the techniques to determine *K*_f_ from infrared spectroscopy and calorimetric measurements and includes the development of additional hydrogen bond acceptance scales.^36–38^

In this context, Laurence et al.^39^ established the p*K*_BHX_ scale, developing and using a database that was initially composed of 1164 HBAs covering a vast chemical space of organic molecules focusing on drug design and medicinal chemistry applications. The p*K*_BHX_ scale depends on the decadic logarithm of *K*_f_, determined experimentally by Fourier transform infrared spectroscopy (FTIR) in CCl_4_ at 25 °C according to eq 2. In this database, the p*K*_BHX_ values span from −0.96 to 5.46, and the HBAs are categorized into five groups: very weak, weak, medium, strong, and very strong. Specifically, very weak HBAs correspond to p*K*_BHX_ values less than −0.7, weak HBAs fall within the range of −0.7 < p*K*_BHX_ < 0.5, medium are characterized by p*K*_BHX_ values between 0.5 and 1.8, strong HBAs are identified by p*K*_BHX_ values between 1.8 and 3.0, and very strong HBAs are defined by p*K*_BHX_ values greater than 3.0.

2 $$pK_{BHX}=\log_{10}K=\log_{10}\frac{\left[HBA\cdot \cdot \cdot HBD\right]}{\left[HBA\right]\left[HBD\right]}$$

### 2.2 Natural Bond Orbitals

Natural bond orbital (NBO) analysis can often provide an idealized natural Lewis structure (NLS) representation for a given wave function.^40–44^ In some cases, such as resonance, hypervalence, or metallic bond, a single Lewis representation cannot accurately describe a molecular system. Nevertheless, the NBO produces a basis set ({Ω_*i*_}, eq 3) composed of localized Lewis-type “donor” NBOs (occupied orbitals, {Ω_*i*_^(L)^}), as one center lone pair of electrons or a two-centered bonding orbital that maximize overall electron occupancy, as well as non-Lewis-type “acceptor” NBOs (vacant orbitals, {Ω_*j*_^(NL)^}) as antibonding orbital.^40^

3 $$\left\{\Omega_{i}\right\}=\left\{\Omega_{i}^{\left(L\right)}\right\}+\left\{\Omega_{j}^{\left(NL\right)}\right\}$$

The NBO analysis quantifies the extent of electron delocalization by computing all possible interactions between donor and acceptor orbitals to account for deviations from the NLS. The second-order perturbation theory can estimate the orbital stabilization energy (*E* ^(2)^) resulting from donor–acceptor interactions according to eq 4, where *q*_*i*_ is the occupancy of the donating orbital; ε_*i*_ and ε_*j*_ are the energy of donor and acceptor NBO, respectively, and F­(*i*, *j*) is the Fock matrix element between NBO orbitals *i* and *j*, which is proportional to the overlap integral. Therefore, the *E* ^(2)^ value tends to be higher when the overlap between the orbitals is greater or when the energy difference between the orbitals is smaller. The *E* ^(2)^ value reflects a more intense interaction between donor and acceptor orbitals.^40^ Owing to these characteristics, NBO analysis was chosen as the basis for developing the ML models for predicting p*K*_BHX_.

4 $$E^{\left(2\right)}=\Delta E_{ij}=q_{i}\frac{F{\left(i,j\right)}^{2}}{\varepsilon_{j}-\varepsilon_{i}}$$

## 3 Results and Discussion

Defining the attributes used for modeling is necessary to build machine learning models, and generating and processing these models involves several stages, beginning with the collection and preprocessing of raw data. In chemoinformatics, several software programs can transform a molecule into a vector that computers can process. These vectors can contain topological, physicochemical properties, and molecular fragments, and it is also possible to add properties based on density functional theory quantum mechanics calculations as features for predicting desired molecular properties. This work uses descriptors derived from the NBOs to explore different machine learning regressor models for predicting hydrogen bond acceptance based on the p*K*_BHX_ scale, as they are properties widely explored in the literature to describe the charge-donating and charge-accepting capacity in molecules.^45^

### 3.1 Natural Bond Orbital Descriptors

It is well-known that intermolecular interactions, specifically, the formation of hydrogen-bonded complexes, involve electrostatic interactions and consequently charge transfer (CT) processes.^17,46–48^ These have been previously studied using the NBO by orbital stabilization energy (*E* ^(2)^).^45,49^ In the study by Zou et al., the behavior of NH_3_ as both a halogen- and hydrogen-bond acceptor was explored as a molecular model system.^45^ They demonstrated a linear correlation between interaction strength and the *E* ^(2)^ values resulting from the interaction of the lone pair donor orbital of NH_3_ (i.e., LP­(N)) with the σ* orbital across a series of halogen- and hydrogen-bonded acceptors (LP­(N)···BD*­(R–X) for halogen and LP­(N)···BD*­(R–H) for hydrogen bond donors).^45^ Additionally, they showed that there is a linear correlation between the amount of charge transferred and the *E* ^(2)^ parameter, highlighting the relationship between these two phenomena with the information derived from NBO analysis.^45^ Inspired by this approach, we aim to investigate a potential correlation between *E* ^(2)^ and the hydrogen bond acceptance values from the p*K*_BHX_ scale applying machine learning techniques. To achieve this, we decided to focus our attention on the electronic processes that take place in the reference HBD of the p*K*_BHX_ scale, the 4-FPh (Figure 1), considering that the CT process from forming a hydrogen bond complex (HBA···HBD) causes deviations from the idealized NLS of the 4-FPh.

**Figure 1:**
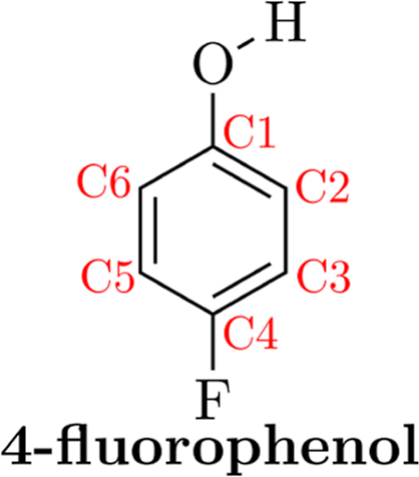
4-Fluorophenol with numbered carbon atoms.

We first computed the *E* ^(2)^ resulting from NBO analysis from the optimized geometry of the 4-FPh. Here, we found eight strong donor–acceptor interactions, i.e., with *E* ^(2)^ greater than 10 kcal mol^–1^, ranging from 19.23 to 34.24 kcal mol^–1^ (Table 1). Such interactions were composed of eight NBOs (Figure 2), including lone pairs (n) from fluorine and oxygen atoms and carbon–carbon π bonds as donor orbitals, and carbon–carbon π* antibonding acting as acceptor orbitals. The occupancy and form of the NBOs that allow us to assign these orbitals are listed in Table S2 in the Supporting Information.

**Table 1:** Orbital Stabilization Energy (*E* ^(2)^, kcal mol^–1^) from Second-Order Perturbation Corresponding to the Most Relevant Donor–Acceptor Interaction in the 4-FPh, by NBO Using CAM-B3LYP/def2-TZV//SMD­(Tetrachloromethane) Level of Theory

donor (*i*)	acceptor (*j*)	*E*^(2)^/kcal mol^–1^
*n*_F_	$\pi_{C_{3}-C_{4}}^{*}$	19.23
$\pi_{C_{3}-C_{4}}$	$\pi_{C_{1}-C_{2}}^{*}$	26.78
$\pi_{C_{1}-C_{2}}$	$\pi_{C_{5}-C_{6}}^{*}$	27.23
$\pi_{C_{5}-C_{6}}$	$\pi_{C_{3}-C_{4}}^{*}$	28.53
$\pi_{C_{5}-C_{6}}$	$\pi_{C_{1}-C_{2}}^{*}$	29.87
$\pi_{C_{3}-C_{4}}$	$\pi_{C_{5}-C_{6}}^{*}$	31.03
$\pi_{C_{1}-C_{2}}$	$\pi_{C_{3}-C_{4}}^{*}$	33.05
n_O_	$\pi_{C_{1}-C_{2}}^{*}$	34.24

**Figure 2:**
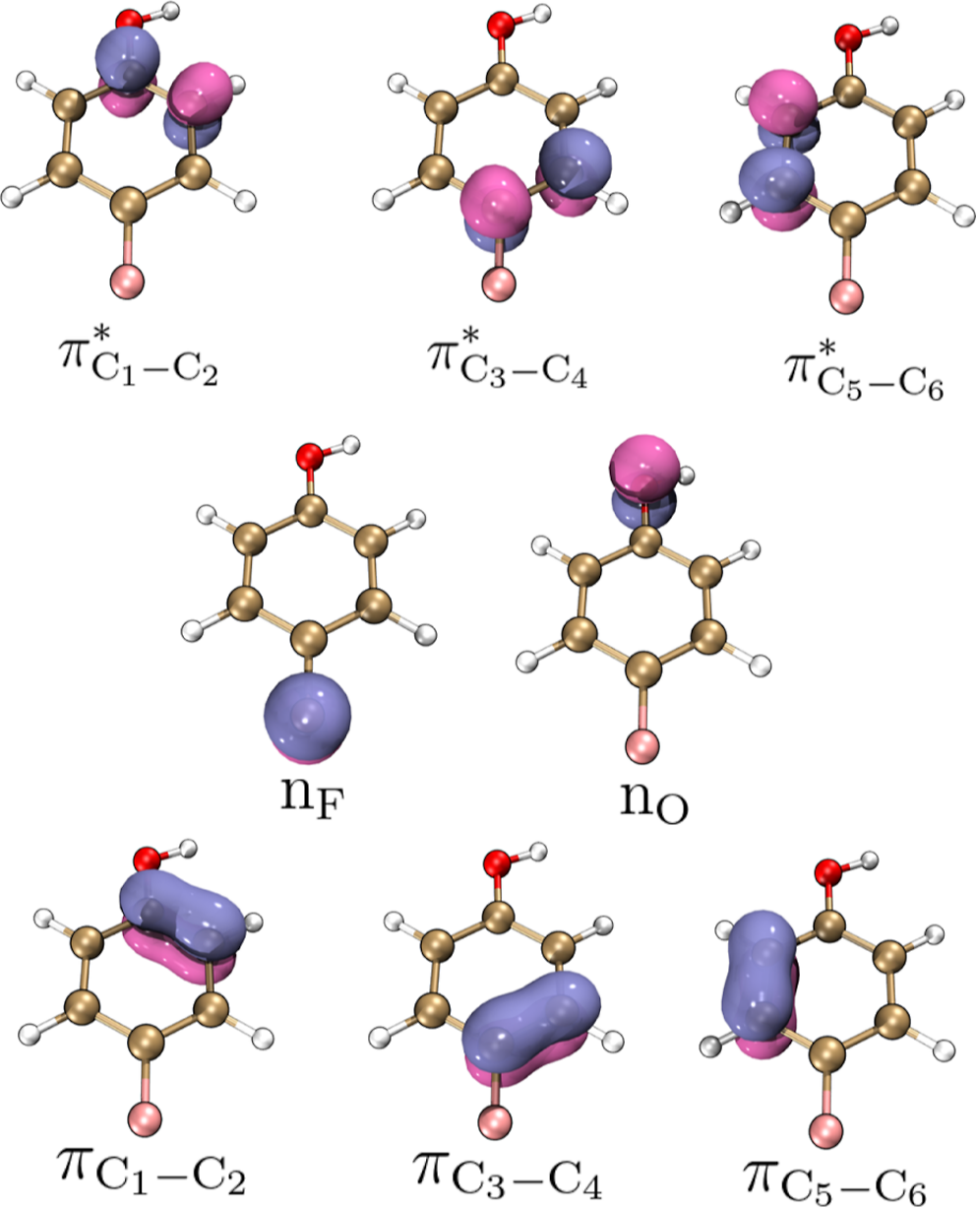
Selected NBO from 4-FPh includes carbon–carbon π* antibonding, the lone pairs (n) located in the fluorine and oxygen atoms, and carbon–carbon π bond.

Interestingly, the NBO donor–acceptor interaction closely relates to the resonance theory description of the electronic delocalization effect. Here, it is possible to assign eight canonical resonance structures for 4-FPh (Figure 3). Notably, the lowest *E* ^(2)^ corresponds to the $n_{F}\to \pi_{C_{3}-C_{4}}^{*}$ interaction, which results in a positive charge on the fluorine atom. Conversely, the highest *E* ^(2)^ correspond to the $n_{O}\to \pi_{C_{1}-C_{2}}^{*}$ interaction, leading to a positive charge on the oxygen atom, indicating that this interaction is the major contribution among all possible resonance structures of 4-FPh. Notably, the intermediate values obtained for *E* ^(2)^ are mainly due to $\pi_{C_{x}-C_{x+1}}\to \pi_{C_{y}-C_{y+1}}^{*}$ delocalization.

**Figure 3:**
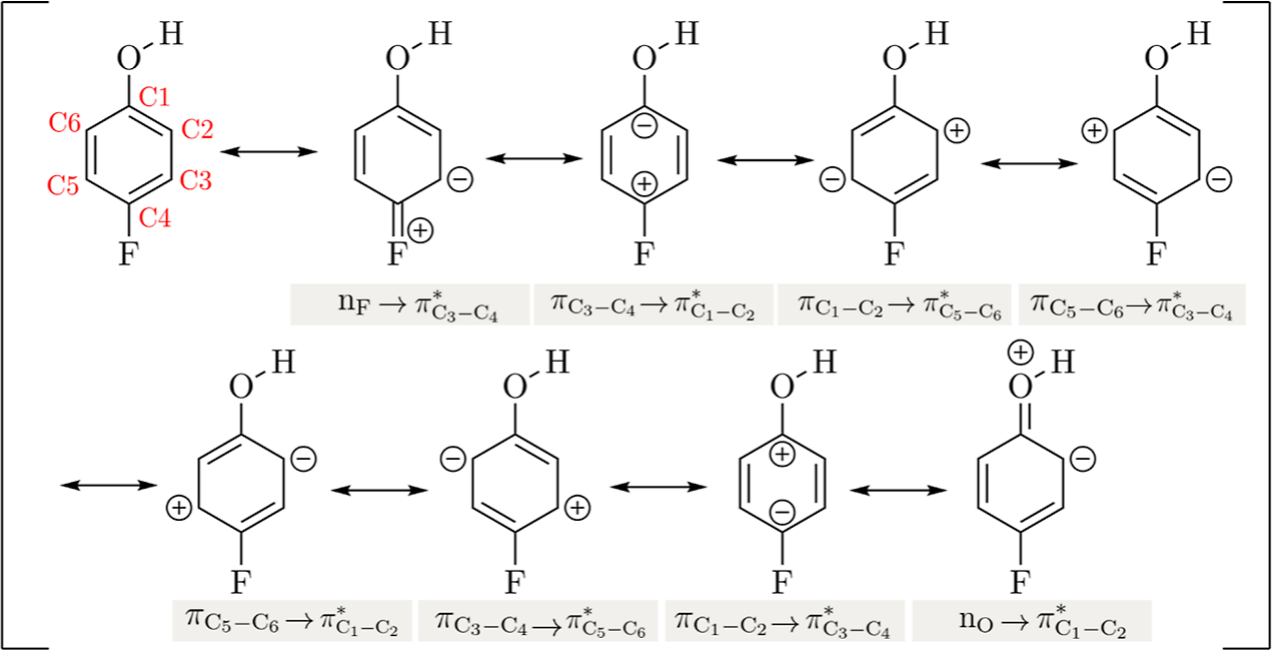
Charge transfer resonance structures associated with NBO donor–acceptor delocalization from 4-FPh, organized according to an increase in *E* ^(2)^ value.

### 3.2 Features Generation

The next step was to compute these eight NBOs in the hydrogen bonding complex following the workflow described in the Method and Computational Details section. For this, we used the data set collected from the literature, which contained 993 p*K*_BHX_ values representing various HBA types. These included oxygen-based groups (carbonyls, ethers, nitro, sulfinyl, phosphoryl), nitrogen-based groups (amines, imines, aromatic nitrogen, nitriles), sulfur-based groups (sulfides, thiocarbonyls), and other functional groups such as aromatics, sulfates, and halogens.^24^

Following the workflow, for the geometry optimization step, we chose the computationally efficient and inexpensive semiempirical GFN2-xTB method, developed by Grimme’s group, combined with an analytical linearized Poisson–Boltzmann (ALPB) implicit solvation model with chloroform. While Laurence’s group conducted the experimental p*K*_BHX_ measurements in tetrachloromethane,^39^ we opted for chloroform due to modeling availability limitations. The GFN2-xTB level of theory has been successfully applied as a surrogate for DFT geometry optimization for building data sets to machine learning models for various chemical property predictions.^50–53^

After geometry optimization and vibrational frequency analysis, 979 p*K*_BHX_ values have been retained. Fourteen molecules were discarded due to either ambiguous SMILES representation or negative frequencies after geometry optimization, indicating a failure to converge to an energy minimum on the potential energy surface. Then, we performed single-point and NBO analysis for all these hydrogen bond complexes. For each HBA···HBD complex, the values of the eight NBO donor–acceptor delocalizations equivalent to those in 4-FPh were computed. Subsequently, these values were used to calculate the Δ*E*_*n*_^(2)^ according to eq 6. Before starting to develop the machine learning (ML) models, we preprocessed the data in these Δ*E*_*n*_^(2)^ to ensure the absence of null values was present, and we standardized the data by subtracting the mean and scaling to unit variance.

Our data set consisted of 979 p*K*_BHX_ values as a target and eight features derived from semiempirical and DFT quantum mechanics calculations for building the ML models. From an exploratory analysis, these p*K*_BHX_ values show an approximately normal distribution with a mean of 1.15 and a range of −1.71 to 4.15 (Figure 4). For the following steps, we randomly partitioned this data set into two subsets. The training set (90%) containing 881 molecules with p*K*_BHX_ values ranging from −1.71 to 4.15, also exhibiting approximately normal distribution with a mean of 1.17 (Figure 4), near the distribution shown in the entire data set, indicating that the training data set represents the chemical information comprehensively; and, the test set (10%) containing 98 molecules with p*K*_BHX_ values ranging from −1.15 to 3.34, also approximating a normal distribution with a mean of 0.97 (Figure 4). This partitioning strategy ensures that the test set remains unseen during the following steps, i.e., hyperparameter tuning and model development, providing a robust assessment of model generalization and accuracy to unseen data; some representative molecules in the training and test data sets are shown in Figure 6.

**Figure 4:**
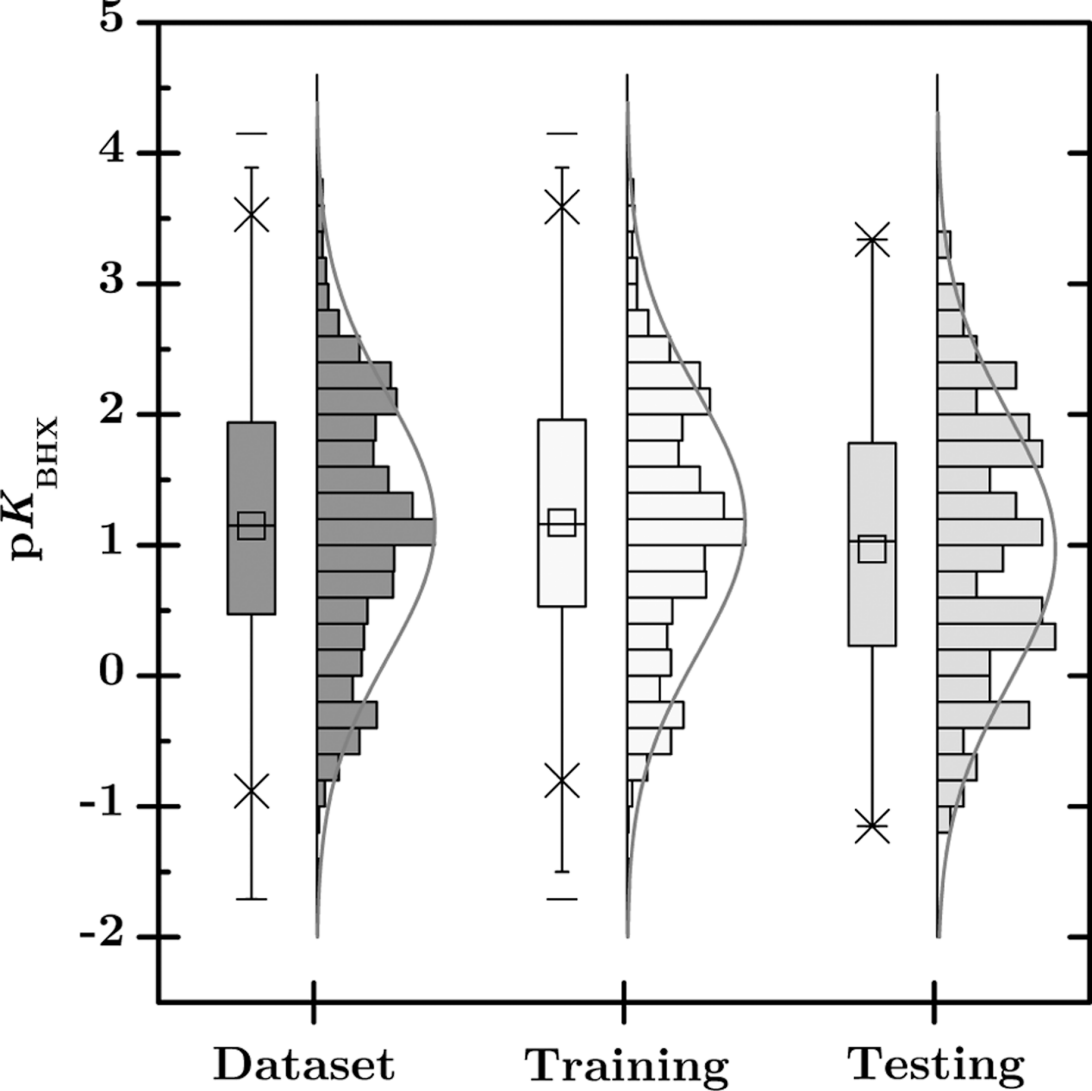
Distribution of p*K*_BHX_ values in the data set, the training and testing data sets. In the boxplot of p*K*_BHX_ values, – represents the maximum and minimum values, × indicates the range between the 1st and 99th percentiles, and □ corresponds to the mean value.

**Figure 5:**
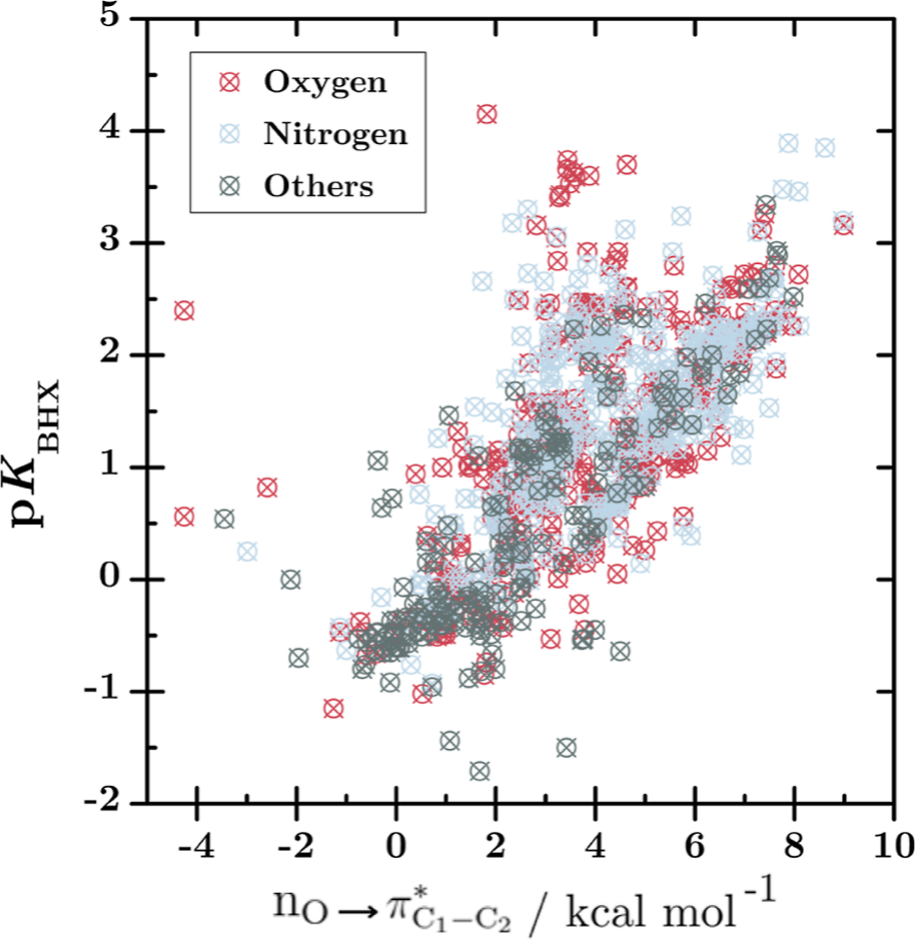
P*K*_BHX_ values from the data set vs the energy of the $n_{O}\to \pi_{C_{1}-C_{2}}^{*}$ interaction. The red, blue, and gray scatters represent the oxygen, nitrogen, and other atoms or bonds as HBAs, respectively.

**Figure 6:**
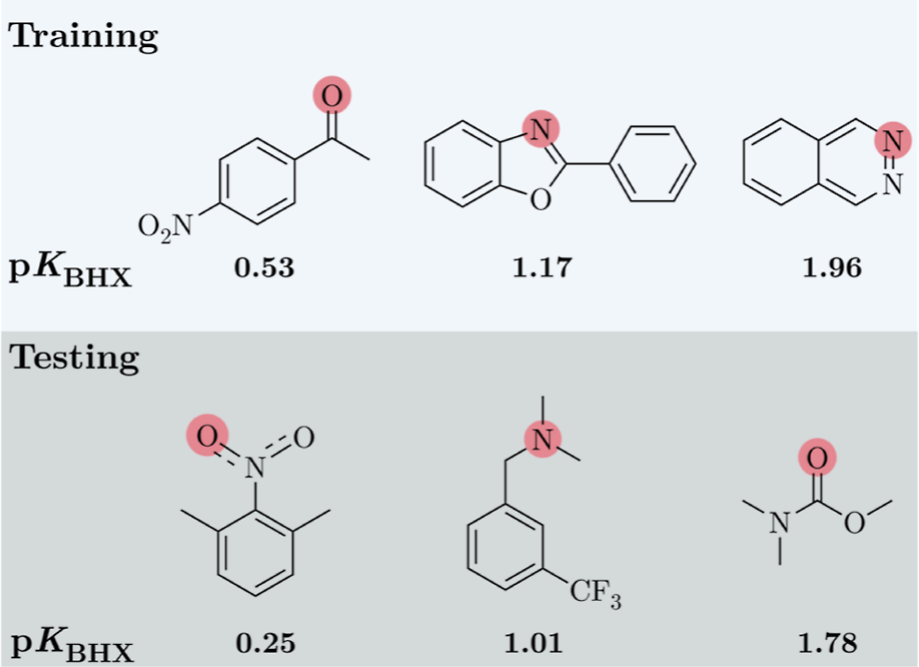
Examples of different HBA in the training and testing data set. The atoms that form hydrogen bonds with 4-FPh are shown in red circles.

Continuing the exploratory analysis of the data set, we find a poor or complete absence of linear correlation of each isolated Δ*E*_*n*_^(2)^ descriptor with the structural nature of the HBAs and, consequently, with the p*K*_BHX_ values. One example of such a poor correlation is shown in Figure 5, considering only the $n_{O}\to \pi_{C_{1}-C_{2}}^{*}$ interaction (for the other interactions see Figure S1). Of the total 979 hydrogen bond complexes, 409 have the nitrogen atom as HBA, 396 have the oxygen atom, and 174 involve other atoms such as halogens or unsaturated or aromatic carbon bonds as HBA (Figure 5). Despite the absence of apparent linear correlation between Δ*E*_*n*_^(2)^ descriptors and the target p*K*_BHX_ values, this does not necessarily imply that there is no relationship whatsoever, since methods such as nonlinear regression based on kernel methods and neural network may be needed to uncover hidden patterns that involve multiple variables.^54–56^

We chose the def2-TZV basis set among the Ahlrichs basis sets of double-, triple-, and quadruple-ζ quality,^57^ considering the balance between computational cost and accuracy, the inclusion of elements below the second row of the periodic table, and the data set size, which contains around 1000 molecules. As already stated, single-point calculations were performed using the Coulomb-attenuated hybrid exchange–correlation functional, CAM-B3LYP.^58^ However, given the potential impact of basis set size on electronic energy, we decided to investigate its effect on the values of our descriptor, Δ*E*_*n*_^(2)^. To this end, we conducted single-point calculations using the def2-SV and def2-QZVP basis sets for the 4-FPh molecule, and for its complexes with four representative HBAs (Figure S2), the ones with the highest and lowest p*K*_BHX_ values and the two HBAs with p*K*_BHX_ values closest to the data set average (Tables S3–S6). We employed the root mean squared error (RMSE, eq 9) to quantify the extent to which the Δ*E*_*n*_^(2)^ values are influenced by basis set size, comparing the Δ*E*_*n*_^(2)^ values obtained with def2-SV and def2-TZV basis sets to the ones calculated with def2-QZVP as reference. For the evaluated cases, we observed RMSE values ranging from 0.10 to 0.40 kcal mol^–1^ for def2-SV and from 0.06 to 0.20 kcal mol^–1^ for def2-TZV, compared to def2-QZVP (Tables S3–S6). Due to these small differences, and considering the above arguments, the def2-TZV basis set is deemed suitable for the used data set; additionally, the chosen methodology is reinforced when we observe the small RMSE from the Δ*E*_*n*_^(2)^ (Tables S3–S6) obtained from geometries calculated with the GFN2-xTB level of theory from these geometries obtained using the CAM-B3LYP­(D3)/def2-TVZP//SMD­(CCl_4_) level of theory; here the RMSE ranges from 0.10 to 1.53 kcal mol^–1^ (Tables S3–S6).

The use of dispersion corrections to account for hydrogen bond interactions is frequently recommended in DFT studies, therefore, we have obtained the geometries with GFN2-xTB and calculated the energies at CAM-B3LYP/def2-TZV level of theory. GFN2-xTB was developed to include multipole electrostatics and density-dependent dispersion contributions.^59^ In fact, over the past decade, xTB has become the method of choice for obtaining geometries of large sets of molecules, due to its reliability and accuracy, including for hydrogen-bonded complexes, as discussed elsewhere.^60–63^ Moreover, CAM-B3LYP is adequate to include dispersion effects; as it can be seen in Table S7, the incorporation of D3 correction in our calculations does not affect the *E* ^(2)^ values, independently of the chosen basis set, def2-TZV or def2-QZVP, given that values were computed at the same geometry.

### 3.3 Machine Learning Models

We evaluated the performance of seven common ML models constructed using *scikit-learn* framework, i.e., Nearest Neighbors (KNN), Decision Tree (DT), Random Forest (RF), Support Vector Machine (SVM), and Multilayer Perceptron (MLP), also with Extreme Gradient Boosting (XGBoost) and CatBoost packages that were trained and used for p*K*_BHX_ prediction. Due to a larger number of hyper-parameters and search space, we optimized these using the Optuna framework,^64^ with the TPE algorithm, a Bayesian optimization to improve model accuracy further, and also to reduce the computational cost compared with other hyper-parameters optimization techniques. The Optuna was set to optimize the hyper-parameters over 500 interactions, employing the RMSE as the optimization standard. The optimized hyperparameters are listed in Table S8 of the Supporting Information.

In this work, we assess the performance of ML models following hyper-parameter optimization using two cross-validation techniques; overall, the hold-out cross-validation (Table 2) proved statistically superior to the K-fold cross-validation applying 5-fold (Table S9). It can be seen that all ML models exhibit good prediction performance, with an *R* ^2^ > 0.80 (Figure 7). The nonparametric method, KNN, yielded the worst accuracy, while improved results could be obtained with decision tree-related models (DT, RF, XGBoost, and CatBoost), which achieved comparable results to the kernel-based SVM. The MLP performs best with RMSE = 0.290, MAE = 0.216, and R^2^ = 0.920 in the test data set (Table 2). The prediction results were as follows in increasing order: KNN < DT < RF < XGBoost < SVM < CatBoost < MLP. In terms of Gibbs free energy change (eq 5), we can estimate from the MAE and RMSE values, that all models have an error of less than 1 kcal mol^–1^ (at 298 K, Figure S3), notably the MLP model with an error of 0.39 and 0.29 kcal mol^–1^ in the RMSE and MAE, respectively. All ML models trained here perform better than a multiple linear regression (Table 2), indicating that a nonlinear model is needed to predict the p*K*_BHX_ values using the descriptors reported here.

5 $$\Delta G=-1.36\;pK_{BHX}$$

**Table 2:** Regression Metrics RMSE, MAE and *R* ^2^ for Models Built with Δ*E*_*n*_^(2)^ Descriptor Using Hold-Out Cross-Validation

hold-out cross-validation			
regressor	RMSE^a^	MAE^a^	*R* ^2^
KNN	0.372 (0.51)	0.250 (0.34)	0.868
Decision Tree	0.341 (0.46)	0.251 (0.34)	0.889
Random Forest	0.309 (0.42)	0.221 (0.30)	0.908
XGBoost	0.307 (0.42)	0.207 (0.28)	0.910
Support Vector Machine	0.304 (0.41)	0.232 (0.32)	0.912
CatBoost	0.304 (0.41)	0.207 (0.28)	0.912
MLP	0.290 (0.39)	0.216 (0.29)	0.920
Multiple Linear	0.471 (0.64)	0.327 (0.44)	0.788

a In parentheses, values calculated in kcal mol^–1^ according to eq 5.

**Figure 7:**
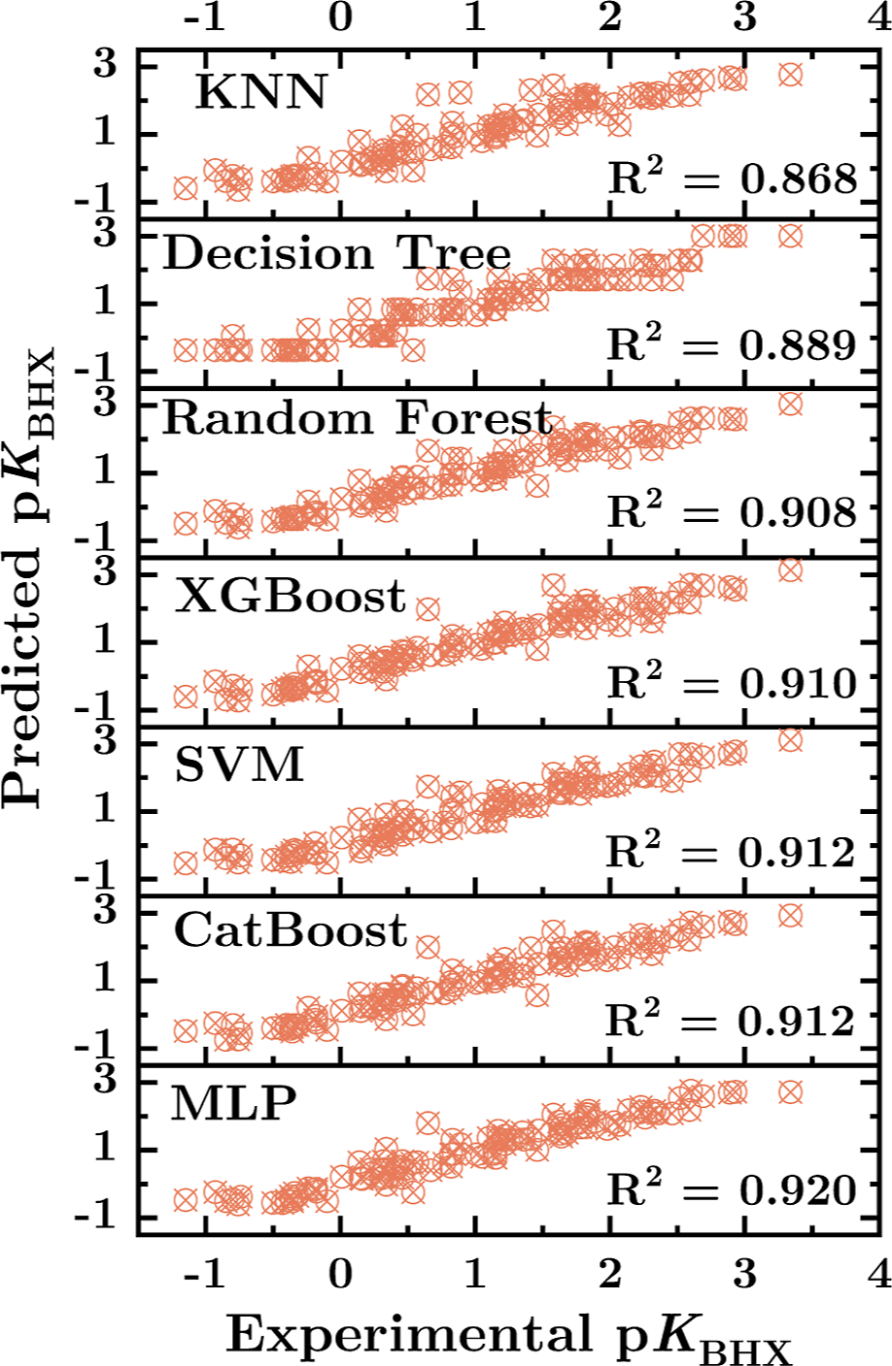
Predicted vs experimental p*K*_BHX_ values from KNN, Decision Tree, Random Forest, XGBoost, SVM, CatBoost, and MLP machine learning models applying Hold-Out cross-validation.

The MLP is a supervised learning algorithm, and for the present work, we have obtained the architecture for this model from Optuna hyper-parameters optimization, comprising one input layer, two hidden layers, and an output layer using backpropagation with a stochastic gradient-based optimizer. A schematic architecture of the MLP implemented here is shown in Figure 8. The input layer contains eight neurons corresponding to the input features, and the two hidden layers contain 62 and 72 neurons in the first and second layers, respectively. All layers are dense, i.e., the neurons in each layer are fully connected with those in the neighboring layer; the rectified linear unit (ReLu) activation function across the hidden layers introduces necessary nonlinearity into the model. The output layer produces the predicted values for the regression task.

**Figure 8:**
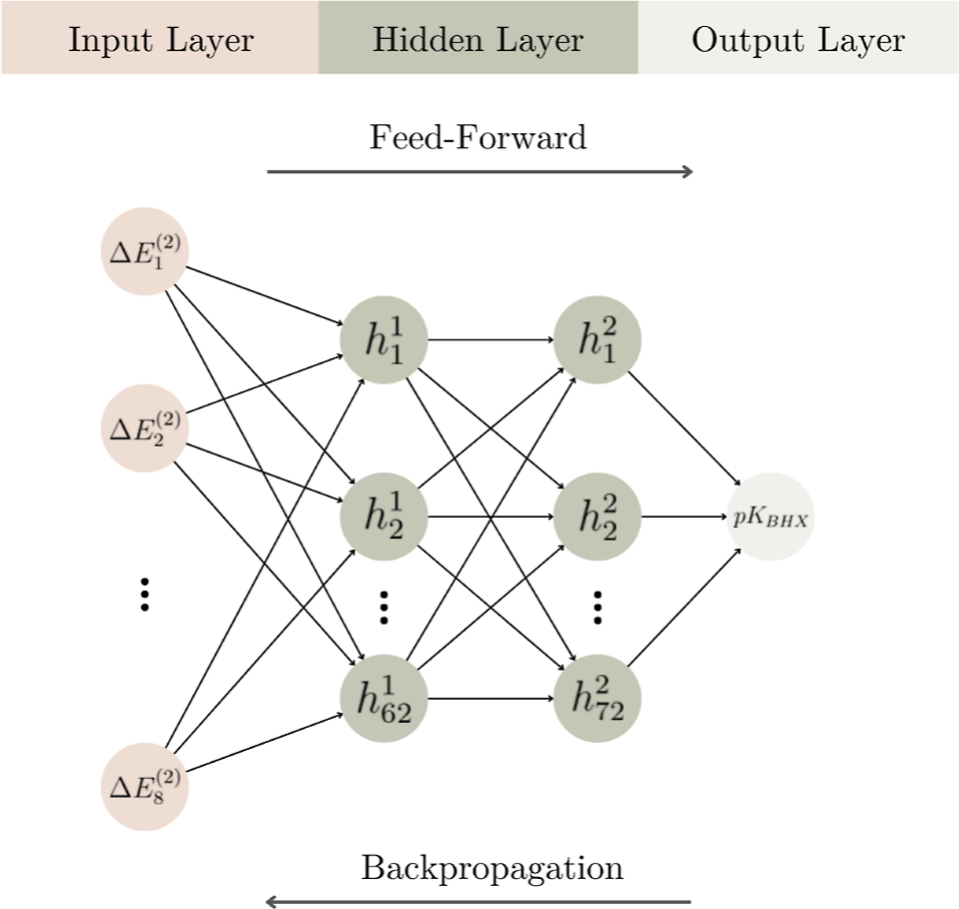
MLP model architecture for p*K*_BHX_ prediction comprising an input layer, two hidden layers, and an output layer.

The predicted p*K*_BHX_ values from the MLP model exhibit a close alignment with the line *y* = *x*, indicating an excellent predictive accuracy (Figure 9A). We also investigate the standardized residual plot to evaluate further the model’s performance (Figure 9B). The alignment of data points along the horizontal regression line indicates that the residuals are unbiased and homoscedastic, exhibiting random scatter with consistent error variance across the independent variable. These findings suggest that the MLP model is adequate for accurately predicting the p*K*_BHX_ values. Nonetheless, it is noteworthy that three points lie outside the 95% confidence interval from linear regression in Figure 9A. We see more clearly these points also exceeding the ± 2σ interval from the standardized residual plot in Figure 9B; these points represent molecules **1**, **2**, and **3**. The observation that different outliers are encountered in the plots for other ML models suggests that the presence of these points is related to the unique generalization capabilities of each model (Figure S4). Specifically, the MLP model identifies three outliers cited above, the KNN model detects six, the CatBoost model has five outliers, DT, RF, and XGBoost each identify four, and the SVM model possesses three outliers.

**Figure 9:**
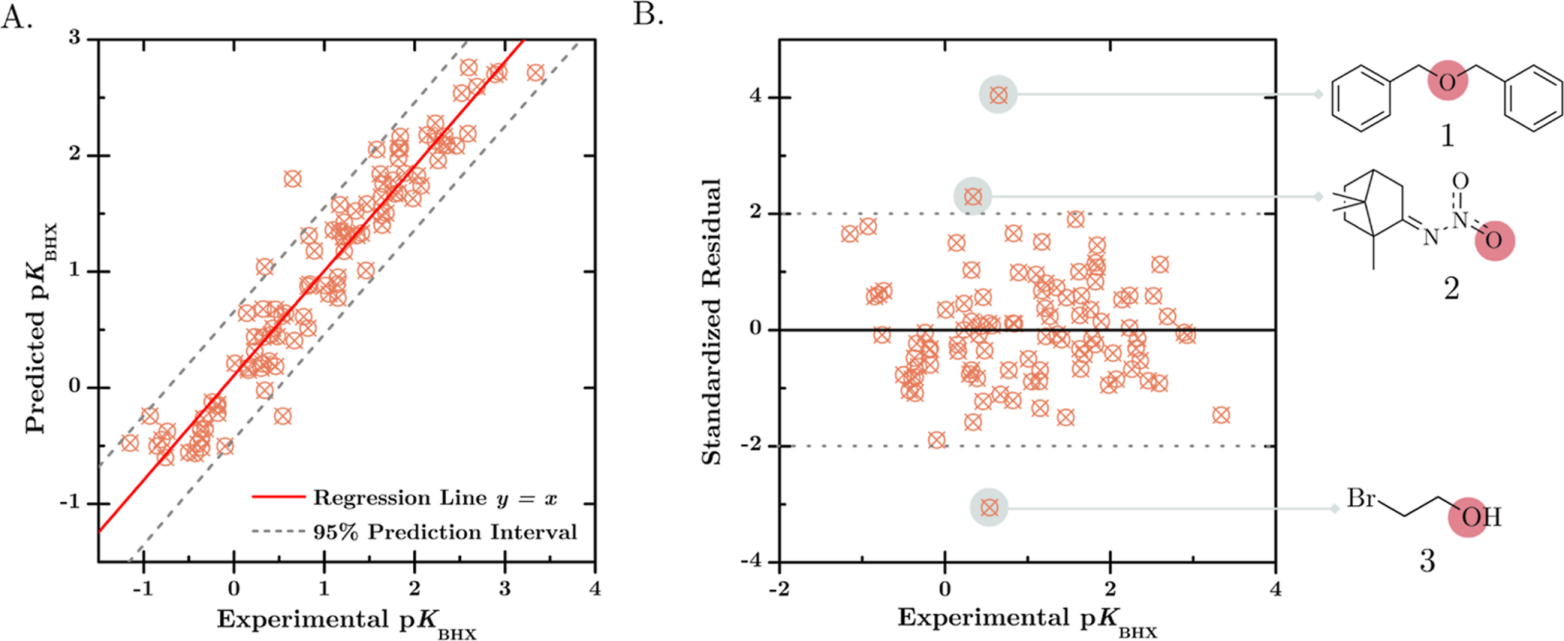
(A) Predicted vs experimental p*K*_BHX_ values from MLP model in the test data set. The red line is the line *y* = *x*, and the 95% confidence interval is the dashed line. (B) Standardized residuals vs experimental p*K*_BHX_ values plot. The horizontal dashed lines define a region where predictions were within ± 2σ residuals intervals; the outlier molecules **1**, **2**, and **3** are shown within gray circles.

Still considering the influence of basis set size on p*K*_BHX_ prediction, we evaluated the p*K*_BHX_ values for four representative HBAs (Figure S2) using descriptors calculated with different basis sets and a previously trained MLP model. A clear improvement in regression metrics (Table S10) was observed when transitioning from the smaller basis set (def2-SV) to def2-TVZ. However, a smaller improvement was observed when the much larger basis set def2-QZVP was used. These results can be interpreted in terms of the concept of target similarity introduced by Huang and Von Lilienfeld in the context of machine learning.^65^ Put simply, target similarity proposes that representations that better embody the underlying physical problem perform better in ML models. The small improvement observed when using def2-QZVP and the excellent performance of the model justify the choice of def2-TVZ basis set for computing the molecular attributes.

To further investigate the internal decision-making of the MLP model, we employed SHAP (SHapley Additive exPlanations) analysis to assess the individual contribution of each Δ*E*_*n*_^(2)^ descriptor to the predicted p*K*_BHX_ values (Figure 10). Among the eight features, the $\pi_{C_{3}-C_{4}}\to \pi_{C_{1}-C_{2}}^{*}$ delocalization emerged as the most influential. Interestingly, higher Δ*E*_*n*_^(2)^ values for this interaction, indicative of increased π → π* delocalization within the aromatic ring of 4-fluorophenol, were associated with lower predicted p*K*_BHX_ values. This finding suggests that when complex formation leads to an increase in this specific electronic signature, the resulting hydrogen bond interaction is weak. Such an interpretation supports the view that the model does not rely solely on the absolute magnitude of the perturbation, but rather on specific patterns of electronic redistribution in the donor that reflect the strength of the intermolecular interaction.

**Figure 10:**
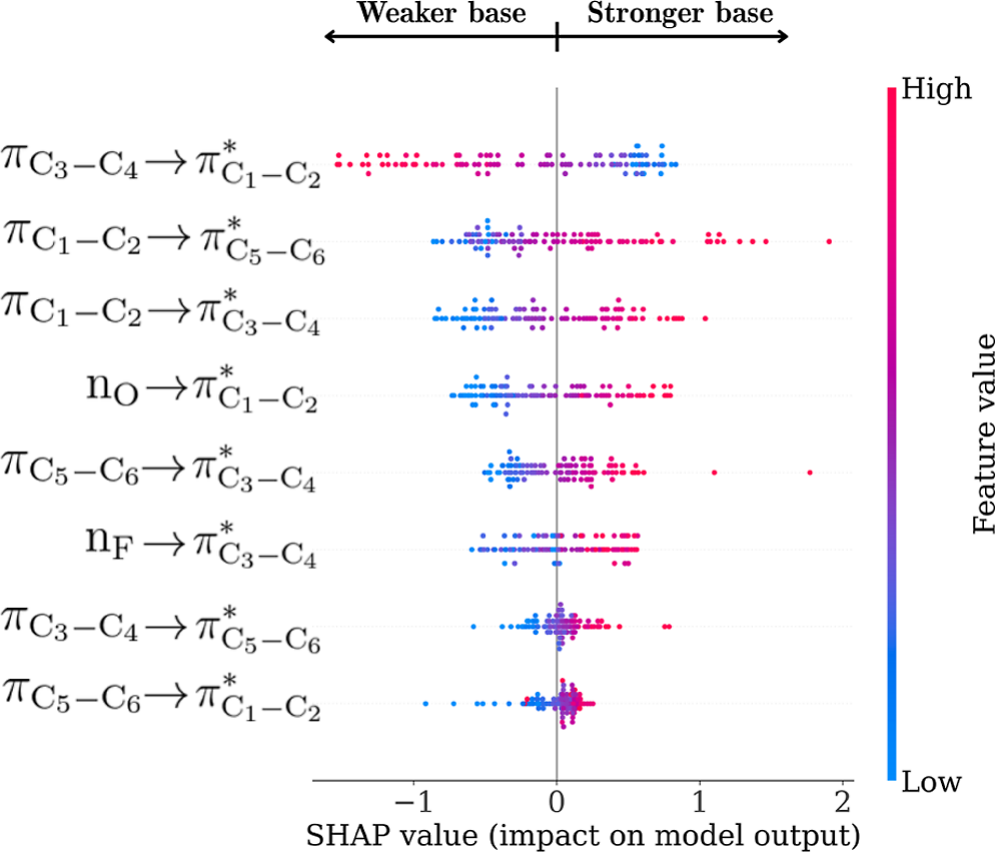
Plot of SHAP for the most important descriptors in predicting the p*K*_BHX_ values using the MLP model.

Interestingly, other features in the SHAP analysis exhibit an opposite trend: higher Δ*E*_*n*_^(2)^ values are associated with increased p*K*_BHX_ predictions, as observed for the n_O_ → $\pi_{C_{1}-C_{2}}^{*}$ and $\pi_{C_{1}-C_{2}}\to \pi_{C_{5}-C_{6}}^{*}$ interactions (Figure 10). These patterns suggest that the model has learned to distinguish between electronic perturbations linked to hydrogen bond stabilization versus those that represent internal stabilization. Increases in Δ*E*_*n*_^(2)^ for the $n_{O}\to \pi_{C_{1}-C_{2}}^{*}$ pathway, for instance, reflect enhanced lone-pair donation from the phenolic oxygen, which is consistent with enhanced charge delocalization and stronger hydrogen bond formation and thus higher p*K*_BHX_ values. Conversely, the negative SHAP direction for the $\pi_{C_{3}-C_{4}}\to \pi_{C_{1}-C_{2}}^{*}$ interaction highlights the role of internal aromatic delocalization in downplaying donor polarization, which is associated with weaker acceptors. These findings underscore the model’s ability to learn chemically relevant patterns beyond simple magnitude correlations.

### 3.4 Comparison with Other Predictive Models of p*K*_BHX_

The H-bond acceptance has been previously studied using various approaches, including quantitative structure–property relationships (QSPR), quantum chemistry, and machine learning (ML) protocols.^24,66^ Among the best-performing models are those based on molecular fragment descriptors and QSPR combined with ensemble modeling, which achieved a RMSE of 0.34–0.37 kcal mol^–1^ and an *R* ^2^ of 0.88–0.91. Bauer et al.,^66^ utilizing data derived from semiempirical and DFT calculations mainly based on the Charge Model 5 (CM5) partial charges and topological information as descriptors, reported an RMSE of 0.91 kcal mol^–1^ and an *R* ^2^ of 0.54 on their test set when applying the Gaussian Process Regression (GPR) model. These authors, when using only first-principles calculations for the determination of hydrogen bonding free energies, obtained an RMSE of 7.25 kcal mol^–1^ compared to experimental free energies; therefore, the ML model with an RMSE of 0.91 kcal mol^–1^ proved to be superior in terms of predictive performance.

Unlike models based solely on 2D descriptors or fragment-based approaches, our method does not suffer from limitations such as a maximum number of heavy atoms, i.e., atoms other than hydrogen, or the inability to predict p*K*_BHX_ values for HBAs whose structural fragments are absent from the training set. While previous studies^66^ have integrated quantum-derived descriptors to overcome the lack of physicochemical information in 2D models, their reliance on large descriptor sets (e.g., over 150 reactivity features) often results in complex, less interpretable models. In contrast, our workflow, which begins with semiempirical geometry optimization and proceeds to DFT-level NBO analysis, achieves broader applicability with only eight well-defined, chemically meaningful descriptors. This compact and interpretable set not only enables accurate predictions across a diverse range of HBAs but also captures essential 3D and electronic features of the full hydrogen bond complex, making our model inherently more robust and generalizable. This highlights how NBO-based descriptors enable a more universal and transferable modeling strategy, improving the generalizability of p*K*_BHX_ prediction across structurally diverse HBAs.

Regarding previously published works, our model achieves equivalent or superior accuracy compared to the QSPR and GPR models, respectively. Given the inherent complexity and black-box nature of certain ML models, using interpretable descriptors, such as those presented in our study, offers a clearer understanding of model predictions. Furthermore, our approach circumvents the need for time-consuming and computationally intensive full geometry optimizations typically required by DFT calculations, making it both efficient and practical for large-scale applications.

## 4 Conclusion

In this work, we investigate the application of natural bond orbital (NBO)-based descriptors to predict the hydrogen bond acceptance p*K*_BHX_ scale using machine learning (ML) regression models. The study focuses on the interactions in hydrogen bond (H-bond) complexes formed by different hydrogen bond acceptors (HBAs) with 4-fluorophenol (4-FPh) as the reference hydrogen bond donor (HBD). The geometries of all H-bond complexes were optimized using the GFN2-xTB semiempirical method, and NBO analysis from density functional theory (DFT) calculations was performed to summarize relevant delocalization interactions in 4-FPh. Their respective orbital stabilization energies (*E* ^(2)^) were used to generate descriptors for our ML models. This study demonstrates that the ML models performed well in predicting p*K*_BHX_ values, with *R* ^2^ > 0.80 and errors in the Gibbs free energy change calculated to be less than 1 kcal mol^–1^ in the test data set. Notably, the MLP model achieved an RMSE of 0.290, an MAE of 0.216, and an *R* ^2^ = 0.920, showing comparable or superior accuracy to other approaches reported in the literature, including models that use only DFT calculations. We also note that despite the low obtained RMSE of 0.290, our models use only eight Δ*E*_*n*_^(2)^ values as molecular descriptors. This reduced number is in stark contrast to the literature regarding ML-based models that frequently use tens to hundreds of molecular descriptors when being developed. In other words, we have shown that Δ*E*_*n*_^(2)^ values encode the H-bond strength, despite no apparent linear correlation with the target property. Taken together, these findings demonstrate that models built on NBO-based descriptors offer a broadly generalizable and chemically interpretable approach for predicting p*K*_BHX_ values across diverse hydrogen bond acceptors. Finally, the excellent accuracy of our ML models paves the way for using chemically interpretable descriptors in future ML studies based on the NBO analysis reported herein.

## 5 Method and Computational Details

### 5.1 Data Acquisition

The initial data set used in this work contains 993 p*K*_BHX_ values, gathered from the literature.^24^ The data set includes the Simplified Molecular Input Line Entry System (SMILES) representation for each molecule, with the hydrogen bond acceptor atom from the HBA molecule marked. The p*K*_BHX_ values comprise both experimental values and, in some cases, approximated values where the experimental determination was challenging.

### 5.2 Feature Engineering

In this initial step, we use several useful RDKit functions (version 2023.09.1)^67^ within a Python 3 environment. We illustrate our workflow (Figure 11) using the *N*,*N*-diethylnicotinamide molecule as an example. To obtain 2D structures, we first applied the MolFromSmiles function in the SMILES encoding, and since HBA molecule SMILES lack explicit hydrogens, we employed the AddHs function. Next, we combine HBA and HBD SMILES using the CombineMols function, generating the desired hydrogen bond complex, and the AddBond function to establish an explicit hydrogen bond between the HBD’s hydrogen atom and the marked HBA atom, producing the final 2D structure. Then, we convert this representation to an SDF file using the MolToMolFile function for subsequent steps.

**Figure 11:**
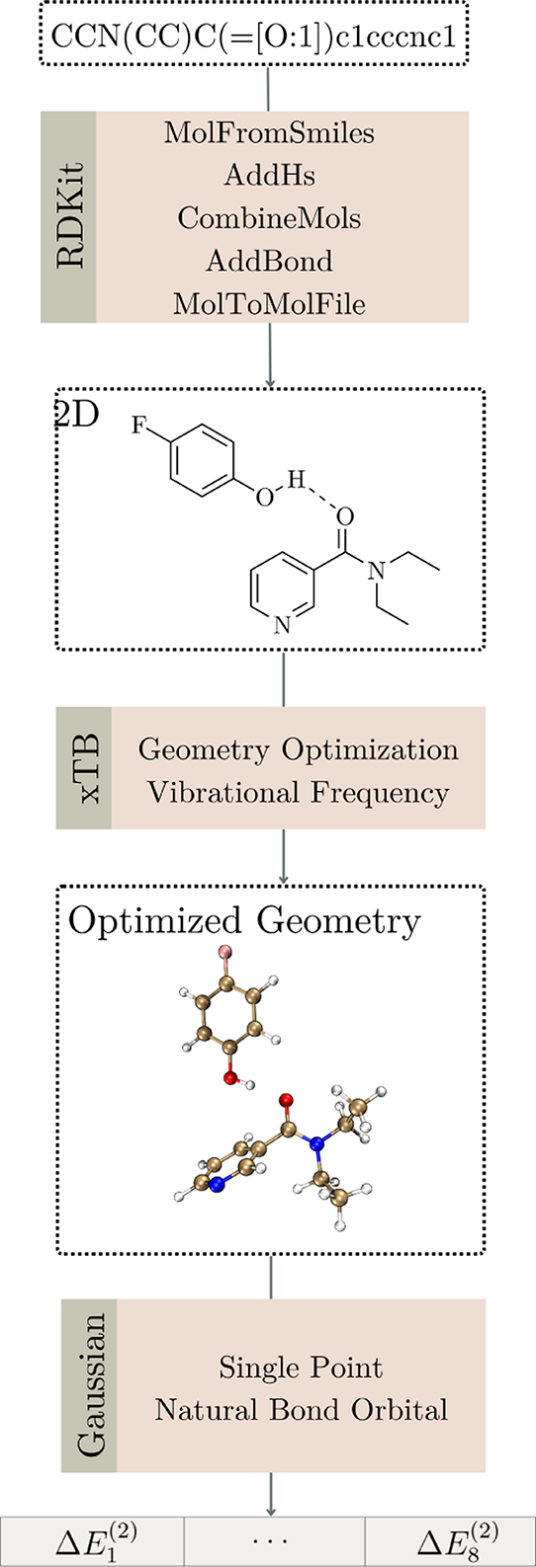
Workflow, from top to bottom, for generating the natural bond orbital descriptors by semiempirical and DFT calculation from SMILES representations.

Following the workflow, we perform geometry optimization from the coordinates extracted from the SDF file with the semiempirical GFN2-xTB method (version 6.5.1),^59,63,68^ using the extreme criteria, followed by a frequency calculation at the same level. We use the analytical linearized Poisson–Boltzmann (ALPB) solvation model with chloroform as implicit solvent.^69^ Using these optimized geometries, we perform density functional theory (DFT)^70^ single-point calculations in Gaussian 09.^71^ This uses the CAM-B3LYP functional,^58^ the def2-TZV basis set,^57^ and the SMD implicit solvent model^72^ with tetrachloromethane. Finally, we used Natural Bond Orbital (NBO, version 3) analysis to calculate the orbital stabilization energies *E* ^(2)^ and compute this from eight relevant NBOs of the 4-FPh molecule within the hydrogen bond complex (*E*_*n*_^(2)^[HBA···HBD], *n* = 1 to 8). Here, we used the same GFN2-xTB method for geometry optimization, followed by single-point calculation at CAM-B3LYP/def2-TZV//SMD­(tetrachloromethane) level of theory to calculate orbital stabilization energies *E*_*n*_^(2)^[4-FPh] for isolated 4-FPh. NBO generation and visualization were performed using Multiwfn and VMD programs, respectively.^73,74^

The final data set used in this work was constructed from Δ*E*_*n*_^(2)^ (eq 6) for each hydrogen bond complex, computed by subtracting the *E*_*n*_^(2)^ from HBA···HBD of the hydrogen bond complex from the corresponding *E*_*n*_^(2)^ of the isolated 4-FPh.

6 $$\Delta E_{n}^{\left(2\right)}=E_{n}^{\left(2\right)}\left[HBA\cdot \cdot \cdot HBD\right]-E_{n}^{\left(2\right)}\left[4-FPh\right]$$

We applied this workflow to all SMILES representations in the initial data set, ensuring that the final data set excluded molecules with inconsistent SMILES representations and negative frequencies after geometry optimization. To construct the machine learning models, we used the Δ*E*_*n*_^(2)^ values as features and the p*K*_BHX_ values as the final target.

### 5.3 Data Preprocessing

Data preprocessing is often a crucial step that can significantly impact the predictive performance of specific machine learning models. It includes handling null values and standardizing features. After verifying the absence of any null values introduced during feature engineering, we utilized the StandardScaler function from the *scikit-learn* ^75^ (version 1.4.0) package to standardize the features. This function is commonly used for centering the data by removing the mean, i.e., each feature has zero mean, and scaling it to have unit variance, as described in eq 7, where the μ is the mean, σ the standard deviation, and *x* the feature value.

7 $$z=\frac{x-\mu }{\sigma }$$

### 5.4 Model Evaluation

There are multiple methods to evaluate a machine learning regression model. To assess the precision of predictive regression models well, we used three different statistical metrics, namely the mean absolute error (MAE, eq 8), the root mean squared error (RMSE, eq 9) and the coefficient of determination (*R* ^2^, eq 10). The MAE calculates the average of the absolute values of the differences between the predicted and actual values; the RMSE calculates the square root of the average of the squares of the differences between the predicted and actual values; and *R* ^2^ quantifies the extent to which the independent variable can account for the variance in the dependent variable. Values near zero for the MAE and RMSE and a value closer to one for the *R* ^2^ indicate superior performance. We estimate these values over *n* samples and calculate them as follows.

8 $$MAE\left(y,ŷ\right)=\frac{1}{n}\sum_{i=1}^{n}\left|y_{i}-{ŷ}_{i}\right|$$

9 $$RMSE\left(y,ŷ\right)=\sqrt{\frac{1}{n}\sum_{i=1}^{n}{\left(y_{i}-{ŷ}_{i}\right)}^{2}}$$

10 $$R^{2}\left(y,ŷ\right)=1-\frac{\sum_{i=1}^{n}{\left(y_{i}-{ŷ}_{i}\right)}^{2}}{\sum_{i=1}^{n}{\left(y_{i}-{\mathrm{y̅}}_{i}\right)}^{2}},\mathrm{y̅}=\frac{1}{n}\sum_{i=1}^{n}y_{i}$$

Here *y*_*i*_ represents the experimental values, and ${ŷ}_{i}$ represents the predicted values of p*K*_BHX_ by the algorithm. We performed this validation using the test set initially generated during the data set processing step and computed it using *scikit-learn*.

### 5.5 Cross-Validation

To avoid overfitting and evaluate and compare the accuracy and robustness of different machine learning models, we employed two strategies with the Hold-Out and K-Fold cross-validation methods. First, we use the Hold-Out method to randomly split the data set with a fixed randomization seed into training and test sets comprising 90% and 10% of p*K*_BHX_, respectively. We used the training set for both hyperparameter tuning and model training, while the test set assessed the final model performance.

We combine the Hold-Out method with K-Fold cross-validation in the second cross-validation strategy. We divide the training data set, generated by the Hold-Out method, into *k* folds (with *k* = 5). We use each fold once as a validation set while combining the remaining *k* – 1 folds for hyperparameter tuning and model training, and this process was repeated *k* times. We evaluate the final model performance using the initial test set generated by the Hold-Out method.

### 5.6 Machine Learning Models

In this study, seven supervised ML algorithms, Decision Tree (DT),^76^ Random Forest (RF),^77^ Multilayer Perceptron (MLP),^75^ K-Nearest Neighbors (KNN),^78^ and Support Vector Machine (SVM),^79^ were implemented using the open source *scikit-learn* library, Categorical Boosting (CatBoost)^80^ and Extreme Gradient Boosting (XGBoost)^81^ packages, within a Python 3 environment to construct regression models. The following sections provide an overview of each algorithm.

Beginning with the Decision Trees model, it is a widely used machine learning algorithm for classification and regression tasks. They are popular for their ease of understanding and visualization. Decision trees work by recursively splitting the data set into subsets based on the most significant feature at each decision node, forming a tree structure where each branch represents a decision path, and each leaf represents an outcome or prediction.

Building on the concept of Decision Trees, Random Forest uses subsets from the training data set to build multiple decision trees, combining them into a single ensemble, where each tree produces its own prediction. The overall output is then obtained by averaging or taking a weighted average of all the trees’ predictions. This strategy allows the Random Forest model to capture more intricate relationships than an individual decision tree.

The Multilayer Perceptron is a feed-forward neural network designed for solving nonlinear problems. It uses a backpropagation algorithm to minimize prediction errors for classification and regression. An MLP consists of a minimum three-layered structure: an input layer, a hidden layer(s), and an output layer. These layers comprise fully connected neurons that apply a nonlinear activation function. The backpropagation allows the network to adjust its weights and biases to minimize the errors between their predicted outputs and the desired targets.^75^

One such lightweight alternative is K-Nearest Neighbor, a nonparametric machine learning technique that assumes similar points are located near each other in feature space, and it is used for both classification and regression tasks. For regression specifically, the KNN model captures local patterns, and the predicted value is the average of the values of *k* nearest neighbors.^78^

The Support Vector Machine is a popular machine learning model for regression and classification. This kernel-based technique is designed to find the optimal hyperplane or decision boundary in high-dimensional space to separate different data points and maximize the margin between them.^79^

Categorical Boosting is a gradient-boosting decision tree (GBDT) algorithm developed by Yandex, which introduces innovations compared to other GBDT models, such as symmetric trees and ordered boosting.^80,82^ It can handle both categorical and numerical features without requiring extensive preprocessing, making it suitable for classification and regression tasks on small and large data sets.^81^

Finally, Extreme Gradient Boosting implements a GBDT technique that combines multiple weak learners into an ensemble method for regression, classification, and ranking tasks. Each new model is trained to correct the errors of the previous models, thereby resulting in a more accurate prediction. It improves upon traditional gradient-boosting techniques by implementing a more regularized model framework, enhancing computational efficiency, and better controlling overfitting.^81^

### 5.7 Hyperparameters Optimization

ML algorithms often possess several hyperparameters that directly influence model generalization and performance. These hyperparameters must be set before training, as the model cannot learn them during the training process. To optimize the performance of each ML regressor model for the p*K*_BHX_ prediction, we used the Optuna package (version 3.5.0),^64^ an open-source automatic hyperparameter optimization framework, to find these, combined with the Tree-structured Parzen Estimator (TPE) algorithm with RMSE as the optimization metric since it is susceptible to outliers in the data set. The optimizer was configured to minimize this score over 500 iterations using the training data set from the Hold-Out method of cross-validation. For a complete list and range of parameters for each ML model, refer to Table S1 from the Supporting Information.

## Supplementary Material







## Data Availability

The data underlying this study are available in the published article, in its Supporting Information, and openly available in GitHub (https://github.com/dmulysses/ml-pkbhx) and DOI 10.5281/zenodo.15602205.

## Notes

Details about the space and range of hyperparameters optimization for all machine learning models; the bond occupancy and composition from NBO analysis; the optimized hyperparameters; the regression metrics RMSE, MAE and *R* ^2^ for models built with Δ*E*_*n*_^(2)^ descriptor using K-Fold cross-validation; effects of the D3 dispersion correction and the def2-SV, def2-TZV, and def2-QZVP basis sets on the representative HBA; the Gibbs free energy change based on the MAE and RMSE errors predicted from ML models; and the predicted vs experimental p*K*_BHX_ values in the test data set, and the standardized residuals vs experimental p*K*_BHX_ values plot, from KNN, DT, RF, XGBoost, SVM and CatBoost ML models (PDF)
SMILES representation comprising the data set, including the calculated *E*_*n*_^(2)^[HBA···HBD] (*n* = 1 to 8); indication if the compound was used in train or test data set; experimental and predicted p*K*_BHX_ values in the KNN, Decision Tree, Random Forest, XGBoost, SVM, CatBoost and MLP models (XLSX)
total-energy-Cartesian-coordenate-complex.xyz: The optimized geometries of the complexes were collected in an .xyz file, all these without imaginary frequencies. The first line contains the number of atoms, the second line contains the SMILES string of the acceptor only (with the mark “:1″ after the atom involved in the H-bond) and the total energy of the complex in Hartree. Starting from the third line, the Cartesian coordinates of the complex geometries are listed (XYZ)
