# Supplementary material for: Developing a Machine Learning Model for Hydrogen Bond Acceptance Based on Natural Bond Orbital Descriptors
Source: J Org Chem. 2025 Jul 7;90(28):9776–88. doi: 10.1021/acs.joc.5c00724 (PMC12281572; doi:10.1021/acs.joc.5c00724)
Supplement: Supplementary file 1 [file jo5c00724_si_001.pdf]

Supporting Information

for

Developing a Machine Learning Model for Hydrogen Bond Acceptance Based on Natural Bond Orbital Descriptors

Diego Ulysses Melo,[†,‡] Leonardo Martins Carneiro,[†,‡] Mauricio Domingues Coutinho-Neto,[†] Paula Homem-de-Mello,[†] and Fernando Heering Bartoloni[*,†]

†*Centro de Ciências Naturais e Humanas, Universidade Federal do ABC, Santo André, São Paulo, Brazil, 09210-580*

‡*D.U.M. and L.M.C. contributed equally to this work*

E-mail: fernando.bartoloni@ufabc.edu.br

List of Contents

	Page
**Table S1.** Space and Range of Hyperparameters Optimization for All Machine Learning Models	S3
**Table S2.** Bond Occupancy and Composition from NBO Analysis	S4
**Figure S1.** The pK_{BHX} values from the dataset vs the energy of the $\Delta E^{(2)}$ interactions.	S4
**Figure S2.** Representative HBA used to evaluate the effects of the basis set.	S5
**Table S3-6.** $\Delta E^{(2)}$ Calculated for Representative Compounds	S5-7
**Table S7.** Calculated Orbital Stabilization Energies $E^{(2)}$ from Relevant NBOs and the Electronic Energy of 4-FPh Molecule Calculated with the CAM-B3LYP Functional with def2-SV, def2-TZV, and def2-QZVP Basis Sets; for the def2-TZV and def2-QZVP Basis Set the $E^{(2)}$ and Electronic Energy was also Calculated Including the D3 Dispersion Correction.	S7
**Table S8.** Optimized Hyperparameters	S8
**Table S9.** Regression Metrics RMSE, MAE and R^2 for Models Built with $\Delta E_n^{(2)}$ Descriptor Using K-Fold Cross-Validation	S8
**Table S10.** Regression Metrics for the Test Dataset in the Trained MLP Model Using the $\Delta E_n^{(2)}$ Descriptor Calculated with the def2-SV, def2-TZV, and def2-QZVP Basis Sets	S8
**Figure S3.** Gibbs free energy change based on the MAE and RMSE errors predicted from ML models	S9
**Figure S4.** The predicted vs. experimental pK_{BHX} values in the test dataset, and the standardized residuals vs. experimental pK_{BHX} values plot, from A) KNN, B) Decision Tree, C) Random Forest, D) XGBoost, E) SVM, and F) CatBoost ML models	S10-11

Table S1: Space and Range of Hyperparameters Optimization for All Machine Learning Models

Model	Parameters
CatBoost	**n_estimators**: [100, 1000]; **learning_rate**: [0.001, 0.1, log=True]; **depth**: [1, 10]; **l2_leaf_reg**: [1, 10, log=True]; **grow_policy**: ["SymmetricTree", "Depthwise", "Lossguide"]
Decision Tree	**max_depth**: [2, 10]; **splitter**: ["best", "random"]; **min_samples_split**: [2, 5]; **min_samples_leaf**: [1, 100]; **min_weight_fraction_leaf**: [0.001, 0.5, log=True]; **min_impurity_decrease**: [0.001, 1, log=True]
Random Forest	**n_estimators**: [100, 1000]; **criterion**: ["squared_error", "absolute_error", "friedman_mse"]; **max_depth**: [1, 10]; **min_samples_split**: [2, 10]; **min_samples_leaf**: [1, 5]; **max_features**: ["sqrt", "log2"]
KNN	**n_neighbors**: [8, 15]; **weights**: ["uniform", "distance"]; **metric**: ["euclidean", "manhattan", "minkowski"]
MLP	**n_layers**: [1, 4]; **n_units**: [1, 256]; **l2_reg**: [1e−5, 1e−2, log=True]; **activation**: ["identity", "tanh", "logistic", "relu"]; **solver**: ["lbfgs", "sgd", "adam"]; **alpha**: [1e−5, 0.01]
SVM	**kernel**: ["linear", "poly", "rbf", "sigmoid"]; **C**: [0.001, 5, log=True]; **degree**: [2, 7]; **gamma**: [0.001, 0.01, log=True]; **coef0**: [0.1, 5, log=True]
XGBoost	**n_estimators**: [100, 1000]; **learning_rate**: [0.001, 0.1, log=True]; **max_depth**: [1, 10]; **subsample**: [0.05, 1]; **colsample_bytree**: [0.05, 1]; **min_child_weight**: [1, 20]

Table S2: Bond, Occupancy, and Bond Composition Obtained from NBO Analysis for 4-FPh

Bond	Occupancy	Bond Composition
$\pi_{C_1-C_2}$	1.66252	(46.92%)C_1(p)+(53.08%)C_2(p)
$\pi_{C_3-C_4}$	1.68301	(48.80%)C_3(p)+(51.20%)C_4(p)
$\pi_{C_5-C_6}$	1.70420	(49.29%)C_5(p)+(50.71%)C_6(p)
$\pi^*_{C_1-C_2}$	0.39102	(53.08%)C_1(p)+(46.92%)C_2(p)
$\pi^*_{C_3-C_4}$	0.38194	(51.20%)C_3(p)+(48.80%)C_4(p)
$\pi^*_{C_5-C_6}$	0.34266	(50.71%)C_5(p)+(49.29%)C_6(p)
n_F	1.94192	(100%)F
n_O	1.88687	(100%)O

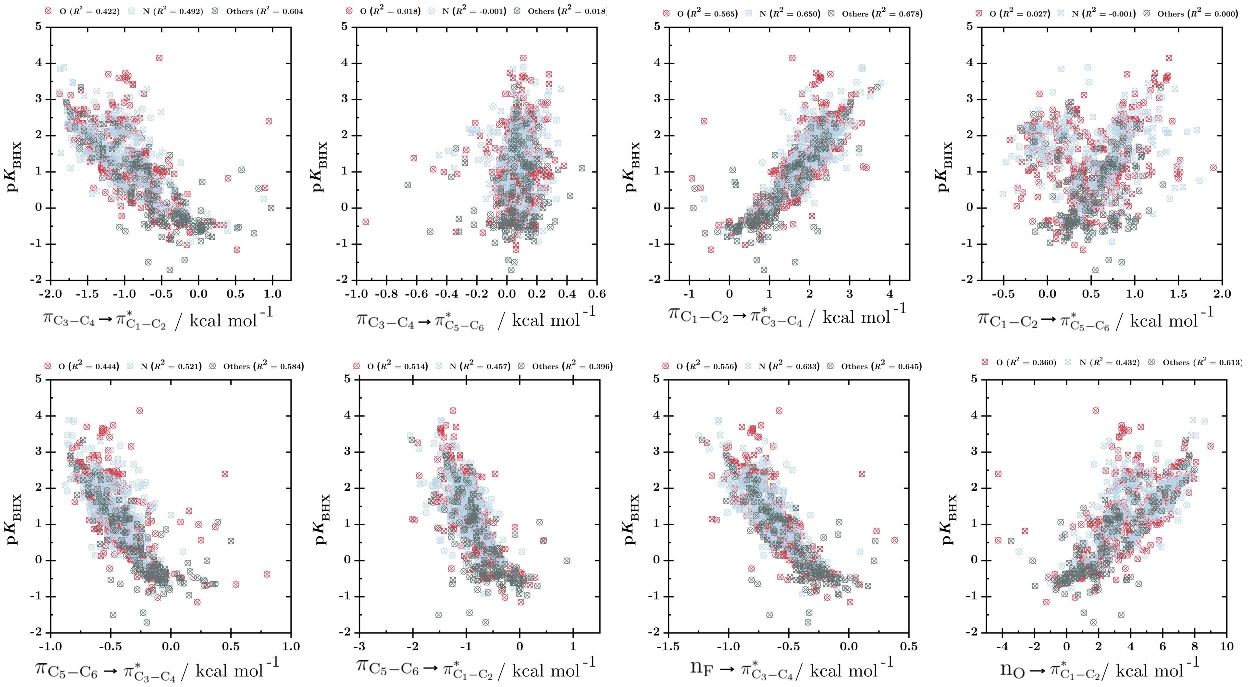

Figure S1: The pK_{BHX} values from the dataset vs the energy of the $\Delta E^{(2)}$ interactions. The red, blue, and gray scatters represent the oxygen, nitrogen, and other atoms or bonds as HBAs, respectively.

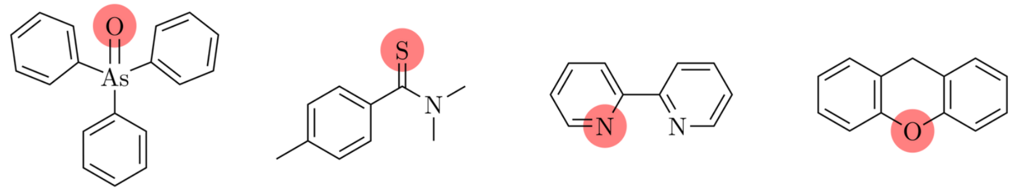

$pK_{BHX} = 4.15$ — [O:1]=[As](c1ccccc1)(c1ccccc1)c1ccccc1

$pK_{BHX} = 1.15$ — CN(C)C(=[S:1])c1ccc(C)cc1

$pK_{BHX} = 1.15$ — c1ccc([n:1]c1)-c1ccccn1

$pK_{BHX} = -1.71$ — cC1c2ccccc2[O:1]c2ccccc12

Figure S2: Representative HBA used to evaluate the effects of the def2-SV, def2-TZV and def2-QZVP basis set. The atoms that form hydrogen bonds with 4-FPh are shown in red circles.

Table S3: The $\Delta E^{(2)}$ (Equation 5) Calculated for the Compound with the SMILES String [O:1]=[As](c1ccccc1)(c1ccccc1)c1ccccc1 (pK_{BHX} = 4.15)

Donor (i)	Acceptor (j)	$\Delta E^{(2)}$ / kcal mol^{-1}			
		def2-SV^a	def2-TZV^a	def2-QZVP^a	def2-TZVP(D3)^b
n_F	$\pi^*_{C_3-C_4}$	−0.47	−0.58	−0.56	−1.20
$\pi_{C_3-C_4}$	$\pi^*_{C_1-C_2}$	−0.31	−0.53	−0.63	−1.20
$\pi_{C_1-C_2}$	$\pi^*_{C_5-C_6}$	1.39	1.39	1.39	1.47
$\pi_{C_5-C_6}$	$\pi^*_{C_3-C_4}$	−0.22	−0.26	−0.31	−0.76
$\pi_{C_5-C_6}$	$\pi^*_{C_1-C_2}$	−1.26	−1.25	−1.30	−1.76
$\pi_{C_3-C_4}$	$\pi^*_{C_5-C_6}$	0.05	0.11	0.09	0.29
$\pi_{C_1-C_2}$	$\pi^*_{C_3-C_4}$	1.43	1.57	1.69	3.11
n_O	$\pi^*_{C_1-C_2}$	1.25	1.82	2.29	5.00
RMSE / kcal mol^{-1}		0.40^c	0.18^c	–	–
		1.53^d	1.32^d	1.15^d	–

^{a}Values of $\Delta E^{(2)}$ from the geometry obtained with GFN2-xTB level of theory; ^{b}Values of $\Delta E^{(2)}$ from the geometry obtained using DFT with CAM-B3LYP/def2-TZVP(D3) level of theory; ^{c}The RMSE using the column def2-QZVP as the reference; ^{d}The RMSE using the column def2-TZVP(D3) basis set as the reference.

Table S4: The $\Delta E^{(2)}$ (Equation 5) Calculated for the Compound with the SMILES String CN(C)C(=[S:1])c1ccc(C)cc1 (pK_{BHX} = 1.15)

Donor (i)	Acceptor (j)	$\Delta E^{(2)}$ / kcal mol^{-1}			
		def2-SV^a	def2-TZV^a	def2-QZVP^a	def2-TZVP(D3)^b
n_F	$\pi^*_{C_3-C_4}$	−0.45	−0.51	−0.49	−0.54
$\pi_{C_3-C_4}$	$\pi^*_{C_1-C_2}$	−0.55	−0.60	−0.69	−0.13
$\pi_{C_1-C_2}$	$\pi^*_{C_5-C_6}$	0.73	0.83	0.80	1.50
$\pi_{C_5-C_6}$	$\pi^*_{C_3-C_4}$	−0.42	−0.45	−0.44	−0.35
$\pi_{C_5-C_6}$	$\pi^*_{C_1-C_2}$	−0.71	−0.79	−0.71	−1.17
$\pi_{C_3-C_4}$	$\pi^*_{C_5-C_6}$	0.24	0.27	0.24	0.34
$\pi_{C_1-C_2}$	$\pi^*_{C_3-C_4}$	1.30	1.35	1.40	1.17
n_O	$\pi^*_{C_1-C_2}$	1.97	2.05	2.25	0.95
RMSE / kcal mol^{-1}		0.12^c	0.09^c	–	–
		0.51^d	0.51^d	0.59^d	–

^{a}Values of $\Delta E^{(2)}$ from the geometry obtained with GFN2-xTB level of theory; ^{b}Values of $\Delta E^{(2)}$ from the geometry obtained using DFT with CAM-B3LYP/def2-TZVP(D3) level of theory; ^{c}The RMSE using the column def2-QZVP as the reference; ^{d}The RMSE using the column def2-TZVP(D3) basis set as the reference.

Table S5: The $\Delta E^{(2)}$ (Equation 5) Calculated for the Compound with the SMILES String c1ccc([n:1]c1)-c1ccccn1 (pK_{BHX} = 1.15)

Donor (i)	Acceptor (j)	$\Delta E^{(2)}$ / kcal mol^{-1}			
		def2-SV^a	def2-TZV^a	def2-QZVP^a	def2-TZVP(D3)^b
n_F	$\pi^*_{C_3-C_4}$	−1.01	−1.14	−1.04	−1.02
$\pi_{C_3-C_4}$	$\pi^*_{C_1-C_2}$	−1.30	−1.32	−1.36	−1.23
$\pi_{C_1-C_2}$	$\pi^*_{C_5-C_6}$	1.30	1.47	1.44	1.52
$\pi_{C_5-C_6}$	$\pi^*_{C_3-C_4}$	−0.63	−0.58	−0.51	−0.56
$\pi_{C_5-C_6}$	$\pi^*_{C_1-C_2}$	−1.63	−2.01	−1.81	−1.77
$\pi_{C_3-C_4}$	$\pi^*_{C_5-C_6}$	0.03	0.00	0.02	0.03
$\pi_{C_1-C_2}$	$\pi^*_{C_3-C_4}$	3.15	3.34	3.13	3.10
n_O	$\pi^*_{C_1-C_2}$	5.75	6.25	5.80	6.01
RMSE / kcal mol^{-1}		0.10^c	0.20^c	–	–
		0.14^d	0.16^d	0.10^d	–

^{a}Values of $\Delta E^{(2)}$ from the geometry obtained with GFN2-xTB level of theory; ^{b}Values of $\Delta E^{(2)}$ from the geometry obtained using DFT with CAM-B3LYP/def2-TZVP(D3) level of theory; ^{c}The RMSE using the column def2-QZVP as the reference; ^{d}The RMSE using the column def2-TZVP(D3) basis set as the reference.

Table S6: The $\Delta E^{(2)}$ (Equation 5) Calculated for the Compound with the SMILES String cC1c2ccccc2[O:1]c2ccccc12 (pK_{BHX} = −1.71)

Donor (i)	Acceptor (j)	$\Delta E^{(2)}$ / kcal mol^{-1}			
		def2-SV^{a}	def2-TZV^{a}	def2-QZVP^{a}	def2-TZVP(D3)^{b}
n_F	$\pi^*_{C_3-C_4}$	−0.27	−0.34	−0.30	−0.32
$\pi_{C_3-C_4}$	$\pi^*_{C_1-C_2}$	−0.20	−0.39	−0.40	−0.12
$\pi_{C_1-C_2}$	$\pi^*_{C_5-C_6}$	0.53	0.55	0.48	0.56
$\pi_{C_5-C_6}$	$\pi^*_{C_3-C_4}$	−0.13	−0.20	−0.20	−0.15
$\pi_{C_5-C_6}$	$\pi^*_{C_1-C_2}$	−0.47	−0.64	−0.51	−0.53
$\pi_{C_3-C_4}$	$\pi^*_{C_5-C_6}$	0.02	0.03	0.05	0.05
$\pi_{C_1-C_2}$	$\pi^*_{C_3-C_4}$	0.66	0.86	0.79	0.85
n_O	$\pi^*_{C_1-C_2}$	1.05	1.68	1.65	1.61
RMSE / kcal mol^{-1}		0.23^{c}	0.06^{c}	–	–
		0.24^{d}	0.19^{d}	0.19^{d}	–

^{a}Values of $\Delta E^{(2)}$ from the geometry obtained with GFN2-xTB level of theory; ^{b}Values of $\Delta E^{(2)}$ from the geometry obtained using DFT with CAM-B3LYP/def2-TZVP(D3) level of theory; ^{c}The RMSE using the column def2-QZVP as the reference; ^{d}The RMSE using the column def2-TZVP(D3) basis set as the reference.

Table S7: Calculated Orbital Stabilization Energies $E^{(2)}$ from Relevant NBOs and the Electronic Energy of 4-FPh Molecule Calculated with the CAM-B3LYP Functional with def2-SV, def2-TZV, and def2-QZVP Basis Sets; for the def2-TZV and def2-QZVP Basis Set the $E^{(2)}$ and Electronic Energy was also Calculated Including the D3 Dispersion Correction

Donor (i)	Acceptor (j)	$E^{(2)}$ / kcal mol^{-1}				
		def2-SV	def2-TZV	def2-TZV(D3)	def2-QZVP	def2-QZVP(D3)
n_F	$\pi^*_{C_3-C_4}$	19.91	19.23	19.23	19.50	19.50
$\pi_{C_3-C_4}$	$\pi^*_{C_1-C_2}$	27.16	26.78	26.78	26.64	26.64
$\pi_{C_1-C_2}$	$\pi^*_{C_5-C_6}$	28.11	27.23	27.23	27.19	27.19
$\pi_{C_5-C_6}$	$\pi^*_{C_3-C_4}$	28.88	28.53	28.53	27.74	27.74
$\pi_{C_5-C_6}$	$\pi^*_{C_1-C_2}$	30.24	29.87	29.87	29.11	29.11
$\pi_{C_3-C_4}$	$\pi^*_{C_5-C_6}$	31.77	31.03	31.03	30.76	30.76
$\pi_{C_1-C_2}$	$\pi^*_{C_3-C_4}$	33.26	33.05	33.05	32.49	32.49
n_O	$\pi^*_{C_1-C_2}$	34.34	34.24	34.24	33.34	33.34
Electronic Energy / Hartree		−406.215320018	−406.569932572	−406.578836513	−406.730222296	−406.739126237

Table S8: Optimized Hyperparameters for All Machine Learning Models

Model	Parameters
CatBoost	**n_estimators**: 518; **learning_rate**: 0.049405517082973915; **depth**: 10; **l2_leaf_reg**: 4.134816151740027; **grow_policy**: "Depthwise"
Decision Tree	**max_depth**: 5; **splitter**: "best"; **min_samples_split**: 3; **min_samples_leaf**: 15; **min_weight_fraction_leaf**: 0.014001513031818463; **min_impurity_decrease**: 0.002364384066963733
Random Forest	**n_estimators**: 393; **criterion**: "absolute_error"; **max_depth**: 10; **min_samples_split**: 2; **min_samples_leaf**: 1; **max_features**: "log2"
KNN	**n_neighbors**: 15; **weights**: "distance"; **metric**: "manhattan"
MLP	**n_layers**: 2; **n_units_0**: 62; n**_units_1**: 72; **activation**: "relu"; **solver**: "adam"; **alpha**: 1.1635523810951026e−05
SVM	**kernel**: "poly"; **C**: 3.7288560084932603; **degree**: 6; **gamma**: 0.0029604914010939115; **coef0**: 3.5821638281807133
XGBoost	**n_estimators**: 185; **learning_rate**: 0.02596425441970596; **max_depth**: 10; **subsample**: 0.8969848974548218; **colsample_bytree**: 0.8607583706114562; **min_child_weight**: 5

Table S9: Regression Metrics RMSE, MAE and R^2 for Models Built with $\Delta E_n^{(2)}$ Descriptor Using K-Fold Cross-Validation

	K-Fold Cross-Validation		
Regressor	RMSE[a]	MAE[a]	R^2
KNN	0.377 (0.51)	0.248 (0.34)	0.864
Decision Tree	0.396 (0.54)	0.287 (0.39)	0.850
Random Forest	0.333 (0.45)	0.236 (0.32)	0.894
XGBoost	0.348 (0.47)	0.224 (0.30)	0.884
Support Vector Machine	0.342 (0.47)	0.255 (0.35)	0.888
CatBoost	0.324 (0.44)	0.220 (0.30)	0.899
MLP	0.340 (0.46)	0.245 (0.33)	0.889

[a]In parentheses, values calculated in kcal mol^{-1} according to Equation 10.

Table S10: Regression Metrics RMSE, MAE, and R^2 for the Test Dataset in the Trained MLP Model Using the $\Delta E_n^{(2)}$ Descriptor Calculated with the def2-SV, def2-TZV, and def2-QZVP Basis Sets

	def2-SV	def2-TZV	def2-QZVP
RMSE	1.085 (1.48)	0.918 (1.25)	0.871 (1.18)
MAE	0.942 (1.28)	0.706 (0.96)	0.652 (0.89)
R^2	0.726	0.804	0.823

[a]In parentheses, values calculated in kcal mol^{-1} according to Equation 10.

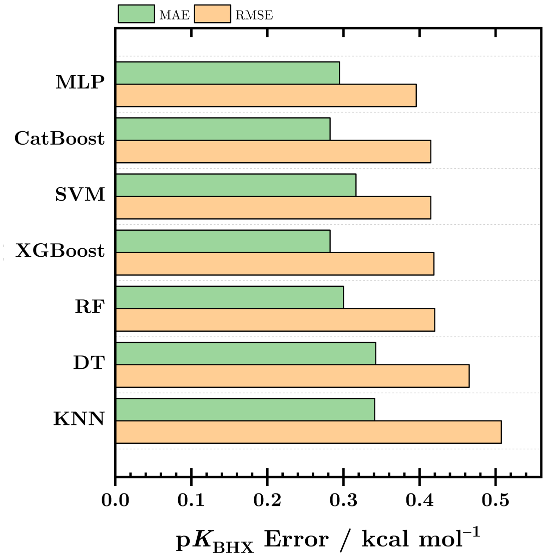

Figure S3: Gibbs free energy change based on the MAE and RMSE errors predicted from ML models.

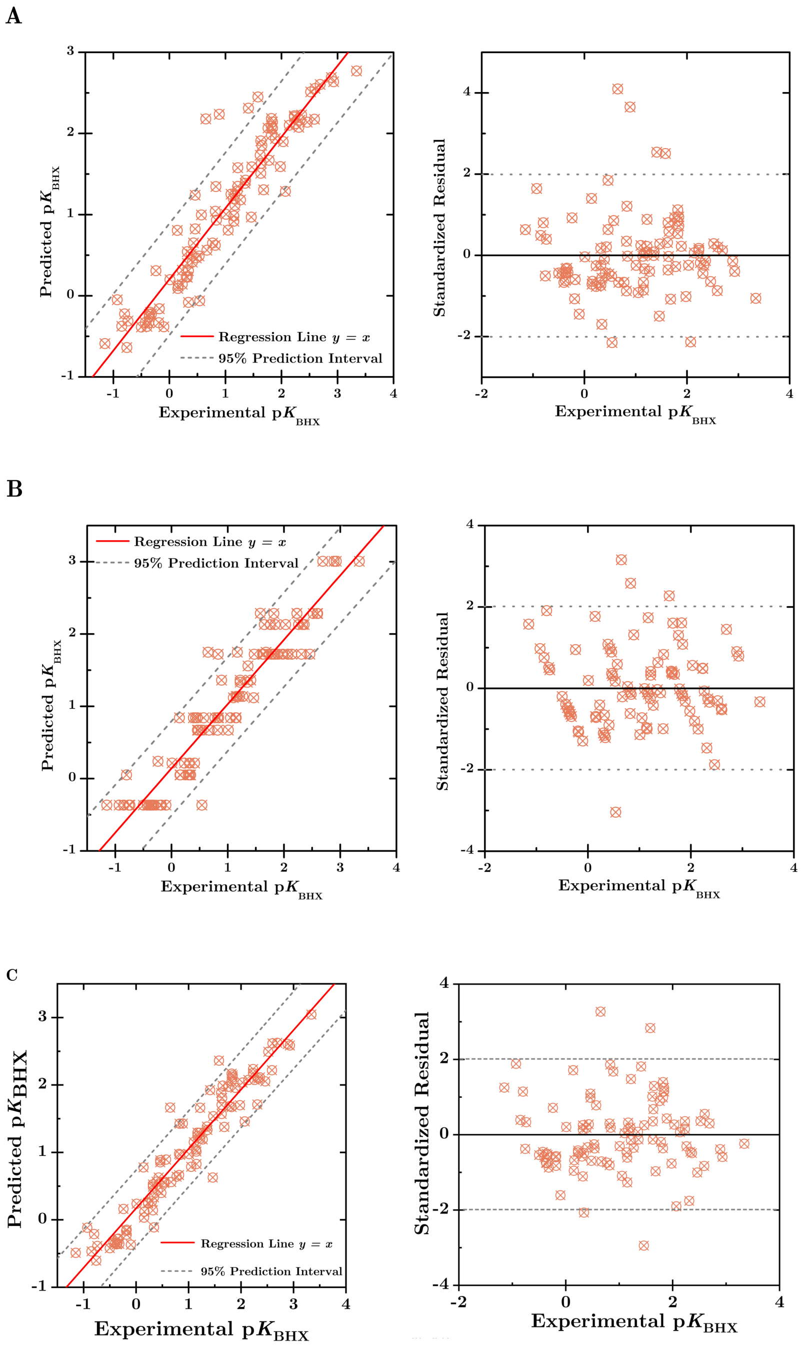

Figure S4: Cont.

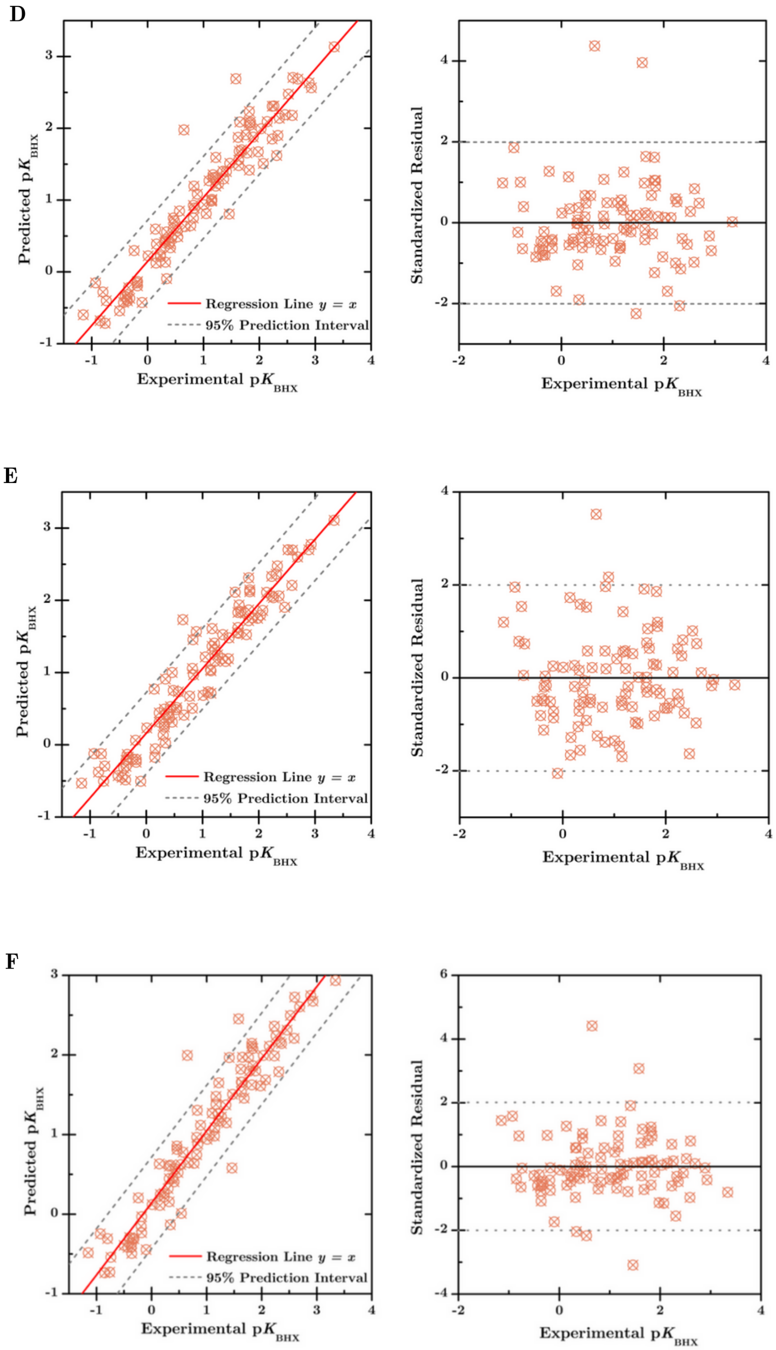

Figure S4: On the left are the predicted vs. experimental pK_{BHX} values in the test dataset, and at the right the standardized residuals vs. experimental pK_{BHX} values plot, from A) KNN, B) Decision Tree, C) RF D) XGBoost, E) SVM and F) CatBoost ML models. The red line in the regression model is the line $y = x$, and the 95% confidence interval is the dashed line. The horizontal dashed lines in the residual plot define a region where predictions were within $\pm 2\sigma$ residual intervals.
